# Haptoglobin, hemopexin, and related defense pathways—basic science, clinical perspectives, and drug development

**DOI:** 10.3389/fphys.2014.00415

**Published:** 2014-10-28

**Authors:** Dominik J. Schaer, Francesca Vinchi, Giada Ingoglia, Emanuela Tolosano, Paul W. Buehler

**Affiliations:** ^1^Division of Internal Medicine, University of ZurichZurich, Switzerland; ^2^Department of Molecular Biotechnology and Health Sciences, University of TorinoTorino, Italy; ^3^Division of Hematology, Laboratory of Biochemistry and Vascular Biology, Center for Biologics Evaluation and Research, Food and Drug AdministrationBethesda, MD, USA

**Keywords:** hemolysis, haptoglobin, hemopexin, CD163, vascular diseases, transfusion, sickle cell disease

## Abstract

Hemolysis, which occurs in many disease states, can trigger a diverse pathophysiologic cascade that is related to the specific biochemical activities of free Hb and its porphyrin component heme. Normal erythropoiesis and concomitant removal of senescent red blood cells (RBC) from the circulation occurs at rates of approximately 2 × 10^6^ RBCs/second. Within this physiologic range of RBC turnover, a small fraction of hemoglobin (Hb) is released into plasma as free extracellular Hb. In humans, there is an efficient multicomponent system of Hb sequestration, oxidative neutralization and clearance. Haptoglobin (Hp) is the primary Hb-binding protein in human plasma, which attenuates the adverse biochemical and physiologic effects of extracellular Hb. The cellular receptor target of Hp is the monocyte/macrophage scavenger receptor, CD163. Following Hb-Hp binding to CD163, cellular internalization of the complex leads to globin and heme metabolism, which is followed by adaptive changes in antioxidant and iron metabolism pathways and macrophage phenotype polarization. When Hb is released from RBCs within the physiologic range of Hp, the potential deleterious effects of Hb are prevented. However, during hyper-hemolytic conditions or with chronic hemolysis, Hp is depleted and Hb readily distributes to tissues where it might be exposed to oxidative conditions. In such conditions, heme can be released from ferric Hb. The free heme can then accelerate tissue damage by promoting peroxidative reactions and activation of inflammatory cascades. Hemopexin (Hx) is another plasma glycoprotein able to bind heme with high affinity. Hx sequesters heme in an inert, non-toxic form and transports it to the liver for catabolism and excretion. In the present review we discuss the components of physiologic Hb/heme detoxification and their potential therapeutic application in a wide range of hemolytic conditions.

## Introduction

Hemolysis with release of free hemoglobin (Hb) and heme occurs in a wide range of disease states and clinical interventions, including genetic and acquired anemias such as sickle cell disease, burns, extracorporeal circulation and massive blood transfusion (Schaer et al., 2013c). Additionally, chemical or recombinant modified Hbs have been evaluated as oxygen therapeutics in several disease states or when blood is not available (Silverman and Weiskopf, 2009). The study of these therapeutic candidates has also provided relevant information regarding the concentration-dependent pathophysiology associated with extracellular Hb in circulation and tissue compartments. Critical reviews provide greater detail concerning their dose dependent toxicity (Buehler et al., 2010).

The primary pathophysiologic effects associated with free Hb/heme are acute hemodynamic instability and acute or chronic tissue injury (Schaer et al., 2013c). The underlying biochemistry of these adverse effects appears to be related to the nitric oxide and oxidant reactivity of free Hb and heme. The ability to counteract extracellular Hb resulting from normal red blood cell turnover and mild hemolysis is one function of haptoglobin (Hp), an α_2_-sialoglycoprotein, which is the primary Hb-binding protein in plasma. Hb-bound Hp targets a specific cellular pathway of clearance through the monocyte/macrophage surface receptor CD163 (Kristiansen et al., 2001). Depending on the extent and frequency of hemolysis, Hp may become depleted, rendering this pathway ineffective. Previous reports from patients with sickle cell disease, spherocytosis, autoimmune hemolytic anemia, erythropoietic protoporphyria and pyruvate kinase deficiency suggest that Hp depletion in plasma occurs prior to the decline of hemopexin (Hx) concentrations (Muller-Eberhard et al., 1968). The disease related consumption of the plasma Hp pool during hemolysis makes Hp a specific clinical marker for intravascular hemolysis (Kormoczi et al., 2006).

Heme released following oxidation of Hb to met-Hb or from heme saturated hepatocytes is bound by albumin and rapidly transferred to Hx, the plasma protein with the highest binding affinity for heme. Hx is another glycoprotein produced by the liver with a plasma concentration of 1–2 mg/ml (Muller-Eberhard et al., 1968). Hx prevents heme's pro-oxidant and pro-inflammatory effects and promotes its detoxification, particularly when Hp concentrations are low or depleted in cases of severe or prolonged hemolysis. Both Hp and Hx are acute-phase proteins, induced during infection and inflammatory states in order to minimize tissue injury and facilitate tissue repair.

The present review will describe the primary mechanisms by which Hp and Hx prevent Hb/heme toxicity prior to monocyte/macrophage-hepatocyte clearance, critically evaluate the difference in genetic phenotype function and describe the rationale for exogenous Hp and Hx as therapeutic proteins for use in hemolytic disease states.

## Mechanisms by which Hp and Hx protect against Hb/heme toxicity

**Figure 1:**
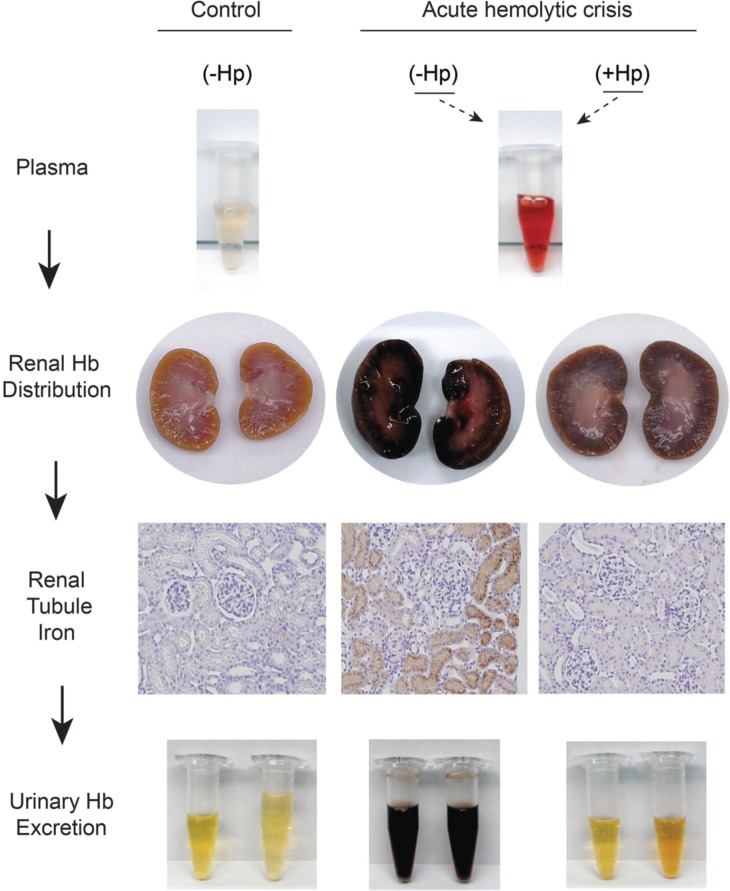
**Sequence of renal plasma clearance of hemoglobin: The sequence represents sustained intravascular hemolysis in a guinea pig model**. The red color of Hb in plasma during hemolysis suggests ferrous Hb accumulation. In the presence of haptoglobin free Hb is sequestrated within the Hb-Hp complex. Processes of Hb-Hp clearance are saturated during massive hemolysis and the complex remains in circulation for an extended period of time, leading to the brownish color or ferric heme in bound Hb dimers, which is a result of auto-oxidation or reaction with NO or other oxidants in plasma. Free hemoglobin readily dissociates into dimers in plasma, leading to extensive glomerular filtration, which is blocked by Hp. The filtration of Hb leads to renal tubule iron deposition (brown) and hemoglobinuria. This is not observed in control animals or when Hp is administered and Hb remains sequestered in the Hb-Hp complex.

Mechanistic research on the *in vitro* and *in vivo* function of Hp and Hx has suggested well defined mechanisms of protection (Schaer et al., 2013c). The best-characterized function of Hp is intravascular sequestration of extracellular Hb following formation of large Hb-Hp protein complexes, a process that prevents extravasation of free Hb into tissues. This effect is particularly evident in the kidneys where oxidative reactions at the heme moiety of Hb lead to globin deposition (hyaline casts), iron overload, lipid peroxidation and renal tubular injury (Qian et al., 2010; Ballarin et al., 2011; Billings et al., 2011) (Figure 1). Similarly, by sequestering heme within a protein complex, Hx prevents heme's ability to intercalate into cell membranes and plasma lipoproteins, where it participates in reactions with organic peroxides, the result of which is the generation of free radicals in localized tissue spaces followed by lipid, protein and DNA oxidation. Additionally, Hx blocks heme activation of immune receptors and vascular inflammatory processes (Belcher et al., 2014). The combined antioxidant and Hb/heme binding characteristics of Hp and Hx allow Hb-Hp and heme-Hx complexes to circulate in the blood in a less toxic form until they can be cleared by their respective scavenger receptors. In the following sections, Hp and Hx will be discussed with an emphasis on tissue protection from the deleterious effects of extracellular Hb and heme.

### Hp and Hx sequester extracellular Hb/heme within the plasma compartment until cellular clearance

Within red blood cells (RBCs), Hb exists as a tetrameric heme containing protein with a molecular weight of 64 kD. Following release from RBCs during intravascular hemolysis, extracellular Hb is in a dynamic tetramer ↔ dimer equilibrium (Ackers and Halvorson, 1974). The dimer fraction of Hb is favored by low Hb concentration and an oxygenated Hb (Fe^2+^) state. The 32-kD Hb dimer readily passes through the glomerulus—if not bound by Hp—and is rapidly cleared by the kidneys (Murray et al., 1961; Andersen et al., 1966; Boretti et al., 2009). Renal filtration is therefore a primary route of clearance for extracellular Hb following Hp depletion. In guinea pigs and dogs, we have previously characterized plasma clearance of extracellular Hb with a circulatory half-life of approximately 10–60 min (Boretti et al., 2009, 2014). This is consistent with earlier findings using radio-labeled Hb in rabbit and dog (Bunn et al., 1969). Hb can also be filtered into the lymphatic system after intravascular injection, and translocates across intact endothelial monolayers both *in vitro* and *in vivo* (Nakai et al., 1998; Faivre-Fiorina et al., 1999; Matheson et al., 2000). The mechanisms underlying this “translocation/extravasation” phenomenon remain unclear, but may involve active transport mechanisms—for example, through the caveolar system, which is also responsible for the controlled transendothelial transport of other plasma proteins such as albumin (Faivre-Fiorina et al., 1999; Komarova and Malik, 2010).

**Figure 2:**
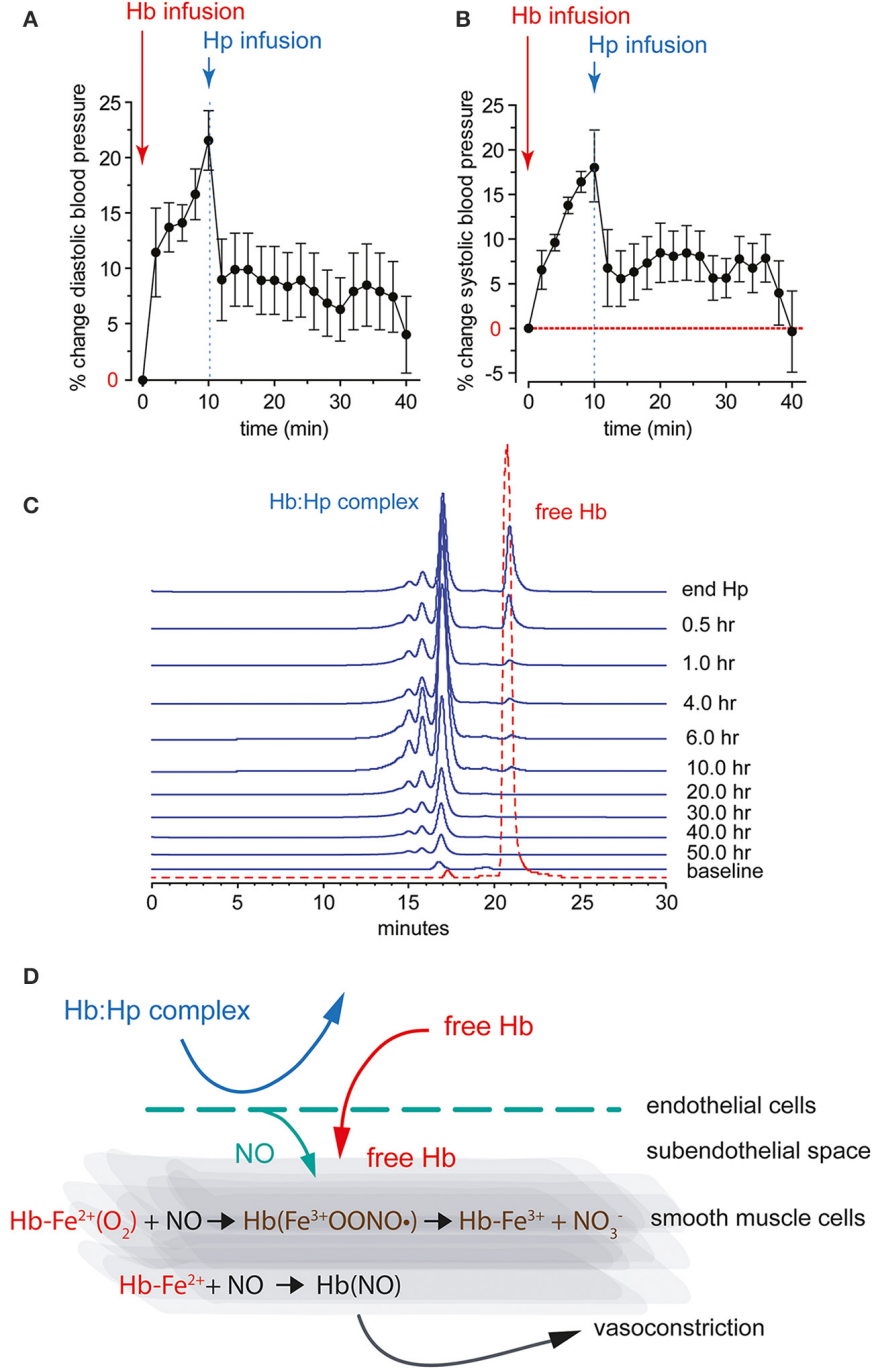
**Diastolic (A) and systolic (B) systemic arterial blood pressure response after bolus infusion of free Hb followed by a bolus infusion of purified human Hp in guinea pigs**. **(C)** HPLC analysis of guinea pig plasma after infusion of free Hb followed by Hp. The traces show free Hb co-eluting with an Hb standard (red). The Hb-Hp complex has a larger molecular weight, as indicated by the earlier elution time. The Hb-Hp complex has a long circulation half-life with detectable levels up to 50 h after infusion in this species. **(D)** NO reactions with free Hb in the vascular wall. Ferrous Hb (Fe^2+^) can react with the vasodilator NO via two reactions: (1) NO dioxygenation of oxy-Hb that generates nitrate (NO^−^_3_) and ferric Hb (Fe^3+^), and (2) iron nitrosylation of deoxy-Hb that occurs by direct iron binding of NO to non-liganded ferrous Hb (Fe^2+^). Both reactions lead to depletion of NO and explain the acute vasoactivity of extracellular Hb, which is attenuated by Hp.

Within the plasma compartment, the abundance of anti-oxidants (i.e., ascorbic acid and urate) stabilizes Hb in a reduced state (Butt et al., 2010). This physiologic function of plasma makes it less likely for Hb to participate in oxidative reactions that lead to rapid met-Hb formation and release of free heme. However, once dimerization of Hb occurs, autoxidation of dimers proceeds rapidly with a rate constant (k_ox_ = 0.24 h^−1^) 16-fold faster than tetrameric Hb (k_ox_ = 0.015 h^−1^) (Griffon et al., 1998). Furthermore, after translocation into the extravascular space, Hb may encounter much harsher oxidative conditions than in plasma. This may be particularly true in diseased and inflamed tissues or after an ischemic insult when oxidants accumulate and an acidic pH is created in local tissue sites. A disconnect of intravascular and extravascular Hb oxidation was observed in animal models of intravascular hemolysis or following infusion of ferrous (Fe^2+^) Hb. While oxidized ferric (Fe^3+^) Hb usually remains below the level of detection in plasma, a large fraction of excreted Hb ultimately appears in the urine in the oxidized ferric state (Boretti et al., 2009). This observation could be extrapolated to other tissues, such as the vascular wall, where Hb(Fe^3+^) may accumulate. The large Hb-Hp complex cannot be filtered by the kidney and *in vitro* translocation across endothelial monolayers is almost completely blocked (Lipiski et al., 2013). The most evident protective function of Hp is therefore the sequestration of extracellular Hb within the antioxidant rich plasma compartment (Figure 2). Following binding, Hp limits Hb's access to susceptible environments where heme release, oxidative processes and NO-consuming reactions are much more likely to occur and may finally lead to the sequelae of Hb toxicity, such as renal failure and vascular injury (Gladwin et al., 2012).

A similar sequestering function is observed with Hx, which is capable of controlling the reactivity of free heme following orientation into Hx's “heme pockets.” By scavenging free heme, Hx prevents heme-mediated oxidative reactions with lipids, proteins, nucleic acids and other biological molecules. Along with Hx, several plasma proteins are able to bind heme, including albumin, lipoproteins, α1-microglobulin, but none of these proteins can efficiently prevent heme intercalation into lipid membranes or block heme's pro-oxidant and pro-inflammatory effects.

### Hp and Hx promote free Hb and heme clearance via receptor targeted pathways

#### Haptoglobin

Hb toxicity largely depends on the rate of hemolysis, tissue oxidant status and clearance capacity. In this network, Hp acts in concert with the plasma's small molecular reducing agents that maintain Hb in a reduced, less reactive ferrous (Fe^2+^) oxidation state (Buehler et al., 2007).

**Figure 3:**
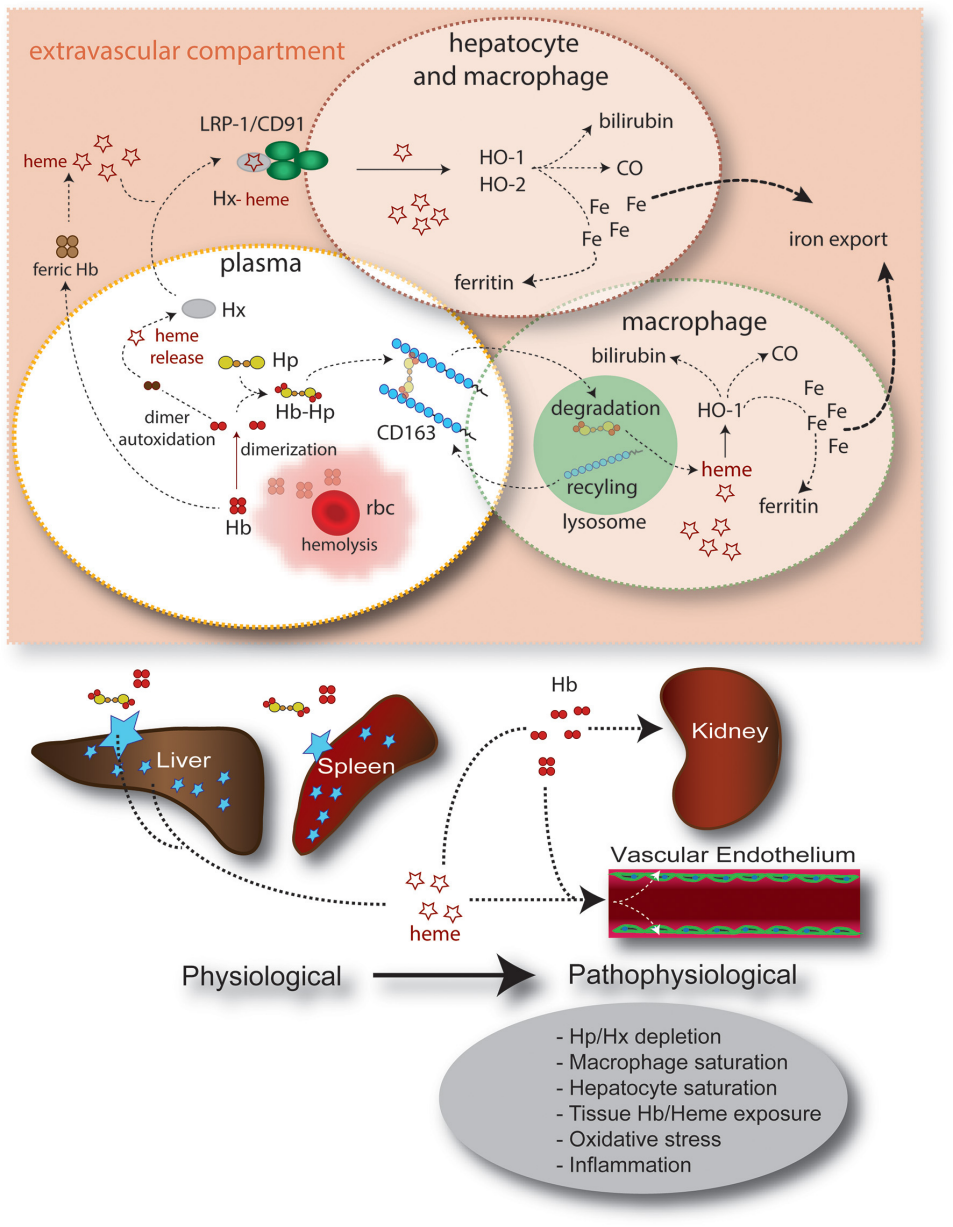
**Summary of intravascular/extravascular Hb/heme clearance and cellular/tissue distribution**. The main components of the Hb/heme detoxification system are haptoglobin (Hp), which sequesters Hb in the oxidatively protected Hb-Hp complex and hemopexin (Hx). The Hb-Hp complex is cleared and metabolized by CD163^+^ macrophages. Alternatively, during more severe hemolysis, free heme—after release from ferric Hb—can be detoxified by the hemopexin (Hx) rescue pathway. In all scenarios heme is finally detoxified by the heme oxygenases, which provide free iron for export by ferroportin or, alternatively, storage in the ferritin complex. The physiologic clearance and detoxification organs for Hb/heme are the liver and spleen, respectively, while the primary targets of Hb/heme toxic activities are the vascular endothelium and the kidney.

In addition to keeping Hb within the antioxidant environment of plasma, Hp plays an essential role in the clearance of Hb and metabolic detoxification of heme (Figure 3). In humans, the scavenger receptor CD163, previously assigned as the macrophage scavenger receptor M130 (Law et al., 1993), has been identified as an endocytosis receptor for Hb-Hp complexes (Kristiansen et al., 2001). CD163 is exclusively expressed by cells of the monocyte-macrophage lineage, with particularly high expression levels in red pulp macrophages of the spleen and liver Kupffer cells (Zwadlo et al., 1987; Bachli et al., 2006). Within the Hb-Hp complex, a high-affinity binding epitope has been identified within the interface region of the Hp β-chain and the Hb α-chain (Madsen et al., 2004; Andersen et al., 2012). Tetrameric Hb can also bind to and is internalized by CD163 in the absence of Hp (Schaer et al., 2006; Buehler et al., 2008). It is unknown whether this lower affinity interaction involves similar regions within Hb and CD163, or whether separate binding structures have independently evolved for Hb and the Hb-Hp complex, respectively (Buehler et al., 2008). Experimental evidence suggests that other Hb and Hb-Hp clearance pathways exist in addition to the CD163 scavenger receptor, and that efficient non-renal Hb clearance mechanisms may be active in the absence of Hp (Murray et al., 1961; Schaer et al., 2006; Etzerodt et al., 2013). However, the nature of these mechanisms requires further studies to be more thoroughly understood.

Inflammatory and anti-inflammatory processes modulate the activity of the Hb-Hp scavenger pathway at multiple levels. Systemic inflammation, particularly if it involves the interleukin (IL)-6 effector pathway, increases expression of Hp in the liver and many parenchymal and non-parenchymal cells. In many species, Hp is one of the most extensively responding acute-phase plasma proteins. IL-6 has also been reported to enhance expression of CD163 on macrophages, suggesting that enhanced Hb sequestration and clearance capacity are general adaptive responses to infection and tissue injury (Buechler et al., 2000). Intriguingly, however, some inflammatory mediators, such as endotoxin and other Toll-like receptor (TLR) agonists or tumor necrosis factor-α (TNF-α) trigger protease-mediated shedding of CD163 from the cell surface of monocytes and macrophages (Droste et al., 1999; Hintz et al., 2002; Moller et al., 2002; Weaver et al., 2006; Etzerodt et al., 2010). This shedding acutely blocks clearance of Hb-Hp complexes by monocytes (Schaer et al., 2007). High levels of soluble CD163 are consequently found in patients with sepsis or more specific macrophage activation syndromes (Schaer et al., 2005; Moller et al., 2006). The physiologic role of this process is unknown, but it implies that complex regulatory mechanisms control Hb detoxification and cellular exposure. Additionally, sCD163 may itself contribute the oxidative detoxification of extracellular Hb and Fcγ-receptor-mediated endocytosis of sCD163-Hb-IgG complexes by monocytes/macrophages, and endothelial cells have been proposed to be an alternative route for Hb clearance (Subramanian et al., 2013).

Of special consideration is the regulation of the Hb clearance system by anti-inflammatory glucocorticoids. In global proteome and transcriptome exploration studies, CD163 was found to be one of the principal glucocorticoid-induced genes in human and mouse macrophages, and patients treated with high-dose pulse glucocorticoids had a significantly enhanced Hb-Hp clearance capacity of their peripheral blood monocytes (Schaer et al., 2001, 2002; Vallelian et al., 2010). Glucocorticoids also act as a transcriptional (co-)activator on Hp synthesis in many species, including human (Marinkovic and Baumann, 1990). In a dog model, a glucocorticoid-supported high Hp level was completely protective against hemoglobinuria and hypertension during an 8-h infusion of free Hb (Boretti et al., 2009). Cumulatively, inflammation and glucocorticoids appear to enhance tolerance against extracellular Hb. In contrast, other drugs such as the antimalarial chloroquine can negatively affect Hb/heme detoxification in macrophages and potentially enhance free Hb toxicity (Schaer et al., 2013b). The potential clinical relevance of these observations—for example, for the treatment of hemolytic anemias—remains to be resolved.

#### Hemopexin

The heme scavenging function of Hx primarily attenuates heme toxicity on the vascular endothelium (Vinchi et al., 2013). While heme easily enters into endothelial cells when bound to albumin, this translocation is completely blocked in the presence of Hx. Heme sequestration within the Hx complex further ensures protection against heme-driven oxidative processes in the extracellular space and it prevents heme-triggered inflammation and adhesion molecule expression.

*In vivo*, the heme-Hx complex is primarily cleared by hepatocytes via receptor-mediated endocytosis (Vinchi et al., 2013). To date, the only known heme-Hx complex receptor is the LDL receptor-related protein 1 (LRP 1), a multi-ligand scavenger receptor (Hvidberg et al., 2005). In addition to hepatocytes, LRP1 is expressed on several other cell types, including macrophages, neurons, and syncytiotrophoblasts. The role of these LRP1 expressing cell types and the possible contribution of alternative receptors in heme-Hx complex clearance remain to be studied.

Some studies have suggested that Hx could be recycled as an intact molecule to the extracellular milieu. However, Hvidberg et al., have shown that most Hx is degraded in lysosomes (Hvidberg et al., 2005). This agrees with the observation that plasma Hx level usually deplete in disorders associated with free heme exposures in both humans and mice.

### Hp and Hx support local NO homeostasis

Depletion of NO, which occurs when the gaseous vasodilator reacts with Hb, explains the acute vasoactivity of extracellular Hb, which is characterized by an acute systemic and, to some extent, pulmonary hypertensive response (Figure 2) (Doherty et al., 1998; Reiter et al., 2002; Gladwin et al., 2004; Olson et al., 2004; Minneci et al., 2005). In experimental animals, consistent acute hypertensive responses can be observed at extracellular Hb levels exceeding approximately 40 μM (heme). The observation that infusion of an Hb-Hp complex lacks the vasoactivity of an equimolar quantity of extracellular Hb has initially suggested that Hp may interfere with Hb's reactions with NO. However, when the NO reactivity of the Hb-Hp complex was measured *in vitro* or in animal plasma, no differences in NO-driven heme oxidation and/or NO consumption were found between free Hb and the Hb-Hp complex, respectively (Boretti et al., 2009). Additionally, Hb-triggered changes in NO metabolite plasma concentrations were not attenuated by Hp treatment at a dose that completely suppressed Hb's hypertensive response (Baek et al., 2012). Therefore, it was speculated that Hp supports local NO bioavailability by keeping the small Hb molecule away from sites of NO production or NO effector functions. Such vulnerable anatomic sites might be the endothelial caveolar system, which represents the primary localization of endothelial NO synthase, the subendothelial space, or the NO responsive contractile smooth muscle cell layers within the wall of arterial resistance vessels (Buehler et al., 2010).

Hx also appears to support vascular NO homeostasis as demonstrated by studies in heme-overloaded Hx-null mice and mouse models of hemolytic disorders (Chiabrando et al., 2014). It is also likely that the beneficial effect of Hx on NO bioavailability is due to heme sequestration away from sites of NO production. Several scenarios could contribute to heme-driven reduction of NO availability. The increased production of reactive oxygen species (ROS) induced by heme may promote NO consumption and formation of peroxynitrite (ONOO-) through the reaction of NO with superoxide (O^·−^_2_). Additionally, NO synthase (NOS), may be uncoupled due to oxidation of the essential cofactor BH4, thus leading to the generation of O^·−^_2_ in place of NO. Accumulation of ONOO- may further contribute to the reduction of NOS activity and the disruption of the NOS dimer.

In summary, although a sparing effect of Hp and Hx on local vascular NO homeostasis is strongly supported, an experimental proof of this concept is still warranted.

### Hp controls oxidative reactions of Hb

Increased tissue concentrations of peroxides [i.e., hydrogen peroxide (H_2_O_2_) or lipid peroxides] occur in many disease states, particularly within the context of inflammatory reactions and during cycles of ischemia followed by reperfusion (Niethammer et al., 2009). The biochemical reactions of Hb with peroxides have been extensively studied *in vitro* and can be summarized as follows: deoxy-Hb reacts with H_2_O_2_ via the following steps: (1) oxo-ferryl Hb [Hb(Fe^4+^ = O)] generation, (2) ferric Hb [Hb(Fe^3+^)] generation, and (3) protein radical generation [·Hb(Fe^4+^ = O)] (Boutaud et al., 2010).

(1) $$\text{Hb}({\text{Fe}}^{2+})+{\text{H}}_{2}{\text{O}}_{2}\;\to \text{Hb}({\text{Fe}}^{4+}=\text{O})+{\text{H}}_{2}\text{O}$$

(2) $$\text{Hb}({\text{Fe}}^{4+}=\text{O})+{\text{H}}^{+}\;\to \text{Hb}({\text{Fe}}^{3+}){\text{OH}}^{-}$$

(3) $$\text{Hb}({\text{Fe}}^{3+})+{\text{H}}_{2}{\text{O}}_{2}\;\to \cdot \text{Hb}({\text{Fe}}^{4+}=\text{O})+{\text{H}}_{2}\text{O}$$

The globin chain free radical shown in reaction 3 can participate in localized amino acid oxidations within Hb (“intrinsic reactions”) or it can transfer to susceptible external molecules such as lipoproteins (“extrinsic reactions”). During the “intrinsic” free radical reactions within Hb, a defined peptide hotspot that is located at the α-globin/β-globin interface becomes the primary site where amino acid oxidation occurs. Among these amino acids is the highly susceptible β-chain Cys93, which can be oxidized to cysteic acid, as well as the α-chain Tyr42, which appears to be an important radical hub within Hb (Jia et al., 2007; Reeder et al., 2008; Pimenova et al., 2010). The cumulative result of these oxidative processes becomes apparent as an unfolding, intermolecular crosslinking and progressive degradation of the Hb molecule (Jia et al., 2007; Vallelian et al., 2008). The oxidized protein can escape clearance by the Hp-CD163 scavenger pathway, and it appears that some Hb degradation products may eventually have direct pro-inflammatory and cytotoxic activities (Buehler et al., 2009; Schaer et al., 2013a). The result of the “extrinsic” radical reactions may be the generation of oxidized lipoproteins, damage to other (non-Hb) proteins and modification of cell membrane components (Miller et al., 1997; Schaer et al., 2013a). Hp binding does not reduce the primary reactivity of Hb with peroxides, a reaction that is generally measured as the initial change in the heme-iron oxidation states (i.e., oxidation of Fe^2+^ → Fe^3+^ → Fe^4+^) during the peroxidative reaction sequence. However, Hp binding limits secondary reactions of these compounds with internal (i.e., globin amino acids) and/or external substrates (Miller et al., 1997; Cooper et al., 2013).

The structural basis of this protection is not yet understood. Hp complex formation may physically limit the transfer of radicals located on the surface of Hb toward external acceptor molecules. Additionally, Hp may limit direct heme pocket access for external substrates. Furthermore, Hb dimerization that is forced by Hb-Hp complex formation may interfere with intra-molecular free radical translocation pathways that normally exist across the Hb dimer interface. Lastly, it has been proposed that Hp may function as a suicidal free radical scavenger within the complex (Pimenova et al., 2010). *In vitro*, the primary result of this oxidative protection becomes apparent as a stabilization of Hb's protein structure (Pimenova et al., 2010).

### Hp prevents heme release from Hb

Heme is an extremely hydrophobic compound that cannot persist as a monomeric soluble molecule under physiologic pH conditions. The term “free heme” is therefore a misnomer, which in fact refers to heme that is not stably located within the heme pocket of a classic heme protein such as Hb or myoglobin. Instead, heme can readily be transferred from ferric (but not from ferrous) Hb to low-affinity heme acceptors (Bunn and Jandl, 1968; Gattoni et al., 1996). The most extensively studied low-affinity heme acceptors are lipoproteins and albumin; however, the group of low-affinity heme acceptors likely involves a very diverse group of soluble and membrane-based proteins and lipids. In the case of lipoproteins, the transferred heme can trigger a lipid-peroxidation cascade, which ultimately results in structural modification of the particle (Balla et al., 1991). Oxidized lipoproteins are cytotoxic and represent one of the principal mediators of Hb toxicity (Nagy et al., 2010; Schaer et al., 2013a). Less understood effects of heme transfer (or “free heme”) are its direct cytotoxic and inflammatory effects (Balla et al., 1993; Belcher et al., 2000, 2003; Wagener et al., 2001; Jeney et al., 2002; Larsen et al., 2010; Fortes et al., 2012). Proposed mechanisms involve stimulation of Toll-like receptors, such as the endotoxin receptor TLR-4 and inhibition of the proteasome, among others (Figueiredo et al., 2007; Lin et al., 2012a; Vallelian et al., 2014).

It is in this context that the fourth known protective activity of Hp appears: the prevention of heme release from ferric (Fe^3+^) Hb (Bunn and Jandl, 1968; Lipiski et al., 2013; Mollan et al., 2014). We have previously learned that ferric Hb (Fe^3+^)—the substrate for heme transfer—does not usually accumulate in plasma, even under severe hemolytic conditions (Baek et al., 2012). It is therefore unlikely that heme transfer from Hb is a relevant process that occurs during hemolysis within the plasma. However, unlike extracellular Hb, the Hb-Hp complex has a slower and saturable clearance, which allows ferric (Fe^3+^) Hb-Hp to accumulate and to reach significant plasma concentrations. In a hemolysis model in guinea pigs, we detected up to 50% of ferric Hb-Hp 24 h after infusion of an Hp bolus for treatment of severe blood transfusion-associated hemolysis. Conceptually, the Hb-Hp complex in plasma may therefore be better compared with a polymerized Hb such as a hemoglobin-based oxygen carrier (HBOC). The intentionally slow clearance rates of HBOCs also allow these substances for significant accumulation of ferric species *in vivo*. Following infusion of most HBOCs there is no barrier against heme release and the oxidative damage that is associated with heme loss may be a significant component of vascular toxicity that accompanies administration of these therapeutic candidates (Natanson et al., 2008). As an evolutionary hypothesis, it is possible that the protective strategy of Hb sequestration within the plasma compartment was only achievable in combination with the sophisticated heme retention and oxidation control capacities of Hp. Only these functions may allow the large Hb-Hp complex to circulate in a less toxic form until cleared. Heme retention and oxidative stability may therefore discriminate the Hb-Hp complex from the adverse event profile of some HBOCs with comparable molecular size and long intravascular retention times (Pimenova et al., 2009; Silverman et al., 2009).

### Hx blocks interactions of free heme with biological molecules

Hx has the highest binding affinity for heme (Kd < 10^−13^ M). Hx sequesters heme in an oxidatively inert conformation in a complex with a 1:1 stoichiometry, until it is cleared by the liver. Heme transfer to more reactive biomolecules is therefore suppressed by Hx. Accordingly, heme-overloaded Hx-null mice show increased oxidative stress in blood vessels, endothelial activation, enhanced inflammation and decreased NO bioavailability (Vinchi et al., 2013). Moreover, Hx-null mice display defective heme accumulation and catabolism in hepatocytes and reduced heme excretion in the bile (Tolosano et al., 1999; Vinchi et al., 2008, 2013).

The work of Belcher et al. (2014) has provided additional insight into how heme sequestration within the heme-Hx complex could attenuate heme-triggered inflammation. Heme is a weak activator of TLR4 and in endothelial cells heme-mediated TLR4 activation promotes Weibel-Palade body (WPB) degranulation, enhanced expression of adhesion molecules and activation of NF-κ B (Belcher et al., 2014). The heme-triggered activation of the TLR4 pathway is blocked by Hx. By similar mechanisms, Hp and Hx may interrupt the synergistic pro-inflammatory and toxic activities of Hb that occur in synergy with HMGB1 and LPS, respectively (Liang et al., 2009; Baek et al., 2014). Other anti-inflammatory effects of Hx may also represent a direct macrophage suppressor effect, which is independent of Hb/heme-triggered signaling (Lin et al., 2012b).

## Haptoglobin and hemopexin in pathology and drug development

### Cardiovascular diseases

Due to its unique anatomic location, the vascular wall appears to be the principal target of free Hb and heme exposure during hemolysis. The diverse Hb/heme-triggered disease processes (i.e., NO consumption, lipid peroxidative processes) are therefore likely to modulate pathologies of the cardiovascular system such as atherosclerosis or typical vascular complications of hemolytic anemias. Oxidized lipoproteins are an established promoter of atherosclerosis and both Hb and heme can promote LDL oxidation (Balla et al., 1991; Belcher et al., 2010; Nagy et al., 2010). Likewise, free heme appears to be an endogenous trigger of endothelium and monocyte/macrophage inflammation, which are both essential in the progression of atherosclerotic lesions (Belcher et al., 2014). Furthermore, heme that is released during microhemorrhage in the vascular wall appears to be a fundamental modulator of the resident macrophage phenotype in atherosclerotic plaques (Boyle et al., 2009, 2012; Kaempfer et al., 2011; Finn et al., 2012). However, despite all this biochemical and cell biologic evidence there is so far no direct experimental or epidemiologic proof that chronic hemolysis or microhemorrhage related Hb release into the vascular wall could directly aggravate atherosclerosis. Accordingly, we do not know whether substitution of Hp or Hx at supra-physiologic levels could attenuate this process. More evidence supports a direct contribution of Hb and heme-triggered reactions to the cardiovascular complication of sickle cell disease, primarily in relation to pulmonary hypertension and the acute chest syndrome. Nitric oxide consumption by free Hb and heme-triggered endothelial inflammatory activation are the principal pathophysiologic components (Rother et al., 2005; Ghosh et al., 2013; Belcher et al., 2014).

### Hp polymorphism and cardiovascular disease associations

In humans, an Hp gene polymorphism exists that determines the three major phenotypes: 1-1, 2-1, and 2-2 (Levy et al., 2010). As a result of a partial intragenic duplication within the Hb α-chain coding region on chromosome 16 (16q22.2), Hp α-chain 2, as opposed to Hp α-chain 1, has two cysteines for disulfide bonding with two other α-chains. Therefore, Hp 2-2 is secreted as a heterogeneous mixture of Hp αβ-chain polymers, whereas the Hp 1-1 phenotype is produced as a homogeneous αβ-dimer. Phenotype 2-1 is a mixed phenotype composed of a range of dimers and polymers. The phenotype distribution varies according to ethnicity; however, in the Western world, it approaches 15% for Hp 1-1, 50% for Hp 2-1, and 35% for Hp 2-2 (Langlois and Delanghe, 1996). The crystal structure of the Hb-Hp complex confirmed earlier biochemical studies predicting that only the non-polymorphic β-chain subunit of Hp interacts with bound Hb αβ-dimer, although no functional role of the α-chain could be inferred from these studies (Andersen et al., 2012). Therefore, per milligram, Hp protein, Hb binding and the physiologic “neutralization” capacity of the three phenotypes should be comparable. The assumption of equal binding has also been confirmed by independent studies in human plasma and with purified Hb-Hp complexes (Delanghe et al., 2000; Lipiski et al., 2013).

In the past, multiple functions of Hp, such as its anti-oxidative and CD163 adaptor functions, have been reported to be phenotype dependent (Asleh et al., 2003, 2005; Levy et al., 2007). Cumulatively, these studies implied that the Hp 2-2 phenotype might be dysfunctional compared to Hp 1-1, with less protective capacity against Hb-triggered pathologies. However, more recently, larger quantities of more standardized and better-characterized Hp proteins became available for experimental studies, including *in vivo* administration studies. These experimental Hp products resulted from industrial efforts to develop phenotype-specific therapeutic Hp products from pooled human plasma. Comparative investigations of two Hp products with predominant Hp 1-1 or Hp 2-2 composition found no significant differences in the intravascular sequestration or renal and vascular short term protection provided by dimeric and multimeric Hp phenotypes in a therapeutic setting of acute intravascular Hb exposure in guinea pigs and dogs (Lipiski et al., 2013; Boretti et al., 2014). Additionally, a range of biochemical studies examining oxidative Hb reactions, prevention of low-density lipoprotein oxidation, and heme retention in the Hb-Hp complex provided evidence of equal protective functions of the dimeric and multimeric phenotypes (Lipiski et al., 2013). Also, NO consumption appears to be identical with the Hb:Hp 1-1 and 2-2 complexes (Azarov et al., 2008; Lipiski et al., 2013).

Epidemiologic studies have also found controversial associations of Hp genotypes with the prevalence and clinical sequelae of atherosclerosis in the general population and in specific patient populations, particularly those with diabetes mellitus. However, more stratified analysis of several large observational studies now convincingly revealed that the Hp 2-2 genotype/phenotype is associated with increased relative risk of coronary artery disease of up to 10-fold in diabetic patients with a glycosylated HbA1c > 6.5 (Cahill et al., 2013). Accompanying mechanistic studies have found higher tissue iron accumulation, additional signs of oxidative damage and a higher number of apoptotic macrophages in aortic atherosclerotic plaques from patients with the Hp 2-2 genotype (Moreno et al., 2008; Purushothaman et al., 2012).

Thus, far, the pathophysiology underlying these associations is unknown. According to studies discussed above, the primary Hb detoxifying functions of Hp 1-1 and Hp 2-2 are comparable and can therefore not easily explain the epidemiologic differences. However, the biologic functions of Hp that have been investigated so far are more representative of the short term protective functions of Hp during acute hemolysis and may not fully reflect longer term antioxidant or immune modulatory functions of the protein that might be more relevant for chronic vascular disease development.

Most epidemiologic studies of phenotype association with cardiovascular disease have been controlled for overall population heterogeneity and for established cardiovascular risk factors; however, these studies were not systematically controlled for differences in Hp plasma concentrations (Cahill et al., 2013). Generally, individuals with the Hp 2-2 phenotype have lower plasma Hp concentrations. Therefore, besides the postulated phenotype dependent molecular functions of Hp, lower Hp concentrations may have an additional impact on vascular disease development in individuals with the Hp 2-2 phenotype (Cid et al., 1993; Shen et al., 2012).

The controversial findings related to Hp phenotype-specific functions and associated disease state risks should certainly stimulate more extensive investigations. However, it now appears that, for therapeutic considerations, the Hp phenotype selection of a product will likely not be a primary determinant of clinical success (Lipiski et al., 2013).

### Disease risk and Hx

A genetic Hx polymorphism has been reported in populations of African ancestry (Kamboh et al., 1993). The biological significance of this polymorphism in hemolytic disorders is merely speculative.

The only available data suggesting a strong association between plasma Hx level and disease risk come from studies evaluating the mouse model of Hx deficiency (Tolosano et al., 1999). Several studies reported a higher susceptibility of Hx-null mice to hemolysis triggered organ damage and heme-driven vascular injury, as discussed below. In other diseases, the impact of Hx has not been directly related to its function as a heme scavenger, likely due to the technical difficulties in quantifying small amounts of “free” heme or possibly due to heme-independent functions of Hx. One such example is related to a regulatory function of Hx in neuroinflammation. Hx deficient mice are more susceptible to the development of experimental autoimmune encephalomyelitis (EAE), the murine model of multiple sclerosis (Rolla et al., 2013). In mice, Hx deficiency favors the differentiation of naïve CD4+ T cells toward Th17 lineage and enhances the stabilization and expansion of memory Th17 cells by IL-23. This is due to a higher disposition of naïve T cells to differentiate toward the Th17 lineage and to a higher production of Th17 differentiating cytokines IL-6 and IL-23 by APCs. These data indicate that Hx may have a negative regulatory role in Th17-mediated inflammation. Interestingly, Hp-null mice subjected to EAE also develop an exacerbated disease and display an increased expression of inflammatory cytokines in the central nervous system, suggesting that Hp and Hx share some anti-inflammatory effects (Galicia et al., 2009). The potential role of the common Hb-heme axis remains to be established.

### Pre-clinical studies of scavenger protein therapeutics

Mouse models of sickle cell disease have a phenotype of chronic heme-driven endothelial activation and dysfunction that could be recovered by repeated Hx administration (Vinchi et al., 2013). These findings highlight a causative role of the free Hb-heme axis in SCD associated vasculopathy. Acute increases in plasma Hb or extracellular heme concentrations can trigger acute vasco-occlusion as well as the acute chest syndrome in SCD mice. Two independent studies have shown that both vaso-occlusion and the acute chest syndrome can be prevented by the infusion of Hp or Hx, respectively (Ghosh et al., 2013; Belcher et al., 2014).

Hemolysis has also been recognized to exacerbate some renal and vascular complications related to the transfusion of stored red blood cells. Retrospective clinical observation studies suggested a link between the storage duration of red blood cells and the incidence of cardiovascular complications such as myocardial infarction, stroke and death. One component of the so-called red blood cell storage lesion is an increased RBC fragility, which may lead to hemolysis post-transfusion. In a guinea pig transfusion model, increased fragility of older RBC could be linked to increased hemolysis in the post-transfusion period, appearance of free Hb in the circulation and Hb-triggered injury in the kidney and vasculature. All these pathologic changes could be prevented by Hp treatment at the time of old blood transfusion (Baek et al., 2012; Lipiski et al., 2013).

Hemolysis with increased free Hb/heme levels and accompanying Hp/Hx depletion can be observed in some patients with severe sepsis. In a mouse sepsis model, Hx administration significantly reduced organ injury and mortality (Larsen et al., 2010), presumably by blocking free heme-mediated oxidative processes and inflammation. In contrast, other mouse studies provided evidence that Hx could even accelerate uncontrolled infection by a poorly defined activity that impairs leukocyte recruitment to the focus of primary infection (Spiller et al., 2011). Observational studies in human patients provided evidence for a positive association of mortality with free Hb release in adults with sepsis (Janz et al., 2013). In this patient cohort, preserved plasma Hp concentrations were significantly correlated with reduced mortality. Whether these data could point to a causative role of free Hb/heme in the progression of human sepsis remains speculative and further laboratory research and better-controlled clinical studies are needed to resolve the role of the Hb/heme-Hp/Hx axis in the control of severe infection and sepsis.

### Potential clinical uses of plasma-derived human Hp and Hx

The rationale for the use of Hp and Hx as therapeutic agents is based on the idea that they act by scavenging circulating Hb and heme, with particular relevance to pathological conditions associated with hemolysis. Central to this concept is the notion that acute and chronic hemolytic conditions are characterized by depletion of the Hb and heme scavengers. Pre-clinical proof-of-concept animal models have demonstrated that Hp and Hx effectively attenuate Hb- and heme-induced vascular and renal pathologies. Based on these preliminary observations, it would appear rational to develop Hp and Hx for therapeutic use (Boretti et al., 2009; Dalton and Podmore, 2011). Plasma-purified Hp has been marketed in Japan since 1985 with primary indications for use in conjunction with extracorporeal circulation, massive transfusion and thermal injury (Schaer et al., 2013c). The primary therapeutic effect in these disease states is protection of the kidneys from Hb-induced toxicity (Hashimoto et al., 1993). Dosing approaches to minimize overall renal exposure to Hb in medically compromised patients are described in a few case reports (Homann et al., 1977; Tanaka et al., 1991; Imaizumi et al., 1994). However, the primary dosing strategy employed in these studies is consistent with a dose-to-effect approach, whereby the Hp dose is increased until amelioration of hemoglobinuria occurs. The Japanese medical communities' use of Hp encompasses a wide range of hemolytic events in approved and off-label disease states in which hemolysis occurs. A new therapeutic protein being considered for approval in the United States and European markets would require safety and efficacy trials in a specific disease state with measurable efficacy end points. For example, in the circumstance of treating a chronic hemolytic anemia, such as sickle cell disease, with repeated Hp and/or Hx doses, a measurable outcome such as significant reduction in vascular complications, reduced hospitalization or duration of hospitalization may be required. As a result, selection of the appropriate disease state(s) to demonstrate measurable improvements in morbidity remains a critical and challenging decision for the drug development process. Pre-clinical studies are still ongoing with the aim of better defining the common and protein-specific functions of Hp and Hx, their disease specific modulatory functions and potential off-target effects. This research may further support well-designed clinical trials translating the protective effects of heme scavengers into clinical progress.

### Conflict of interest statement

The authors declare that the research was conducted in the absence of any commercial or financial relationships that could be construed as a potential conflict of interest.
